# Optimised protocol for monitoring SARS-CoV-2 in wastewater using reverse complement PCR-based whole-genome sequencing

**DOI:** 10.1371/journal.pone.0284211

**Published:** 2023-04-14

**Authors:** Harry T. Child, Paul A. O’Neill, Karen Moore, William Rowe, Hubert Denise, David Bass, Matthew J. Wade, Matt Loose, Steve Paterson, Ronny van Aerle, Aaron R. Jeffries

**Affiliations:** 1 Biosciences, Faculty of Health and Life Sciences, University of Exeter, Exeter, United Kingdom; 2 Analytics & Data Science Directorate, UK Health Security Agency, London, United Kingdom; 3 International Centre of Excellence for Aquatic Animal Health, Weymouth, United Kingdom; 4 Deep Seq, Centre for Genetics and Genomics, Queen’s Medical Centre, The University of Nottingham, Nottingham, United Kingdom; 5 Institute of Infection, Veterinary and Ecological Sciences, University of Liverpool, Liverpool, United Kingdom; University of Helsinki: Helsingin Yliopisto, FINLAND

## Abstract

Monitoring the spread of viral pathogens in the population during epidemics is crucial for mounting an effective public health response. Understanding the viral lineages that constitute the infections in a population can uncover the origins and transmission patterns of outbreaks and detect the emergence of novel variants that may impact the course of an epidemic. Population-level surveillance of viruses through genomic sequencing of wastewater captures unbiased lineage data, including cryptic asymptomatic and undiagnosed infections, and has been shown to detect infection outbreaks and novel variant emergence before detection in clinical samples. Here, we present an optimised protocol for quantification and sequencing of severe acute respiratory syndrome coronavirus 2 (SARS-CoV-2) in influent wastewater, used for high-throughput genomic surveillance in England during the COVID-19 pandemic. This protocol utilises reverse compliment PCR for library preparation, enabling tiled amplification across the whole viral genome and sequencing adapter addition in a single step to enhance efficiency. Sequencing of synthetic SARS-CoV-2 RNA provided evidence validating the efficacy of this protocol, while data from high-throughput sequencing of wastewater samples demonstrated the sensitivity of this method. We also provided guidance on the quality control steps required during library preparation and data analysis. Overall, this represents an effective method for high-throughput sequencing of SARS-CoV-2 in wastewater which can be applied to other viruses and pathogens of humans and animals.

## Introduction

Monitoring the transmission of pathogens in the population is crucial for the prevention and control of infectious disease epidemics. Clinical surveillance of pathogens in individuals is vulnerable to sampling bias, favouring those who pursue healthcare intervention and overlooking asymptomatic infections, as well as being resource intensive and reliant on advanced public healthcare services [1]. Wastewater-based epidemiology provides a complementary method for monitoring pathogens, enabling unbiased population-level surveillance of infections within the catchment area [2]. Environmental surveillance for poliovirus has been deployed for decades [3, 4], allowing effective monitoring of this virus despite its low morbidity rate [5]. Furthermore, viral detection in wastewater using reverse transcription polymerase chain reaction (RT-PCR) has been demonstrated for a range of other pathogenic viruses, including influenza A virus, norovirus, hepatitis A virus and severe acute respiratory syndrome coronavirus 2 (SARS-CoV-2) [6–8], allowing detection of outbreaks prior to recognition in clinical samples [7, 9, 10].

The SARS-CoV-2 pandemic, originating in Wuhan, China in 2019 [11], has been characterised by the successive emergence of variants of concern (VOC) displaying mutations conferring a combination of enhanced infectivity, virulence and immune evasion [12, 13]. Although patient testing methods based on RT-qPCR and lateral-flow immunochromatographic assays have formed the front-line in identification of SARS-CoV-2 cases, genomic surveillance is essential for monitoring the emergence of novel variants and tracing viral transmission patterns in the population, providing crucial data for public health decision-making [14, 15]. However, whole-genome sequencing of SARS-CoV-2 from clinical samples is expensive and often reliant on material from RT-qPCR assays, availability of which is dwindling due to the scaling-back of public SARS-CoV-2 testing. Furthermore, a significant proportion of SARS-CoV-2 cases remain asymptomatic [16, 17], which are likely underrepresented in clinical samples.

After the identification of SARS-CoV-2 RNA in patient stool samples [18, 19], detection of the virus via RT-qPCR in sewage was confirmed [8], indicating the potential utility of wastewater monitoring in SARS-CoV-2 surveillance. Subsequent studies have found that the abundance of SARS-CoV-2 in wastewater correlates with concurrent clinical case numbers [10, 20–22]. Furthermore, genomic wastewater surveillance has been shown to effectively capture lineage dynamics in the population and has proven to be able to detect the introduction of novel variants before their identification in local clinical samples [23–26]. In this way, sequencing SARS-CoV-2 in wastewater has been proposed as an effective way of detecting the emergence of new lineages in the community [26, 27]. Moreover, wastewater monitoring is a cost-effective and unbiased strategy for genomic epidemiology, requiring fewer samples to capture infections at a population-level and thereby reducing the required sequencing costs [28].

Monitoring of SARS-CoV-2 in wastewater routinely involves an enrichment step to concentrate viral RNA from large volumes of wastewater, for which a range of ultrafiltration and precipitation-based methods have been trialled [29, 30]. Once RNA has been extracted and cDNA synthesised, sequencing of SARS-CoV-2 from clinical and wastewater samples has typically been carried out through the generation of tiled amplicons across the genome [31, 32], enabling increased sensitivity compared to metagenomic approaches. This is particularly important for samples with low target concentration, such as SARS-CoV-2 in wastewater [8], and increases the throughput capacity by reducing the per sample library input required and therefore increasing the multiplexing capability. Tiled amplification is typically followed by library preparation for sequencing on Oxford Nanopore Technologies or Illumina platforms, involving the addition of sequencing adapters and barcodes to allow multiple samples to be sequenced simultaneously.

**Fig 1:**
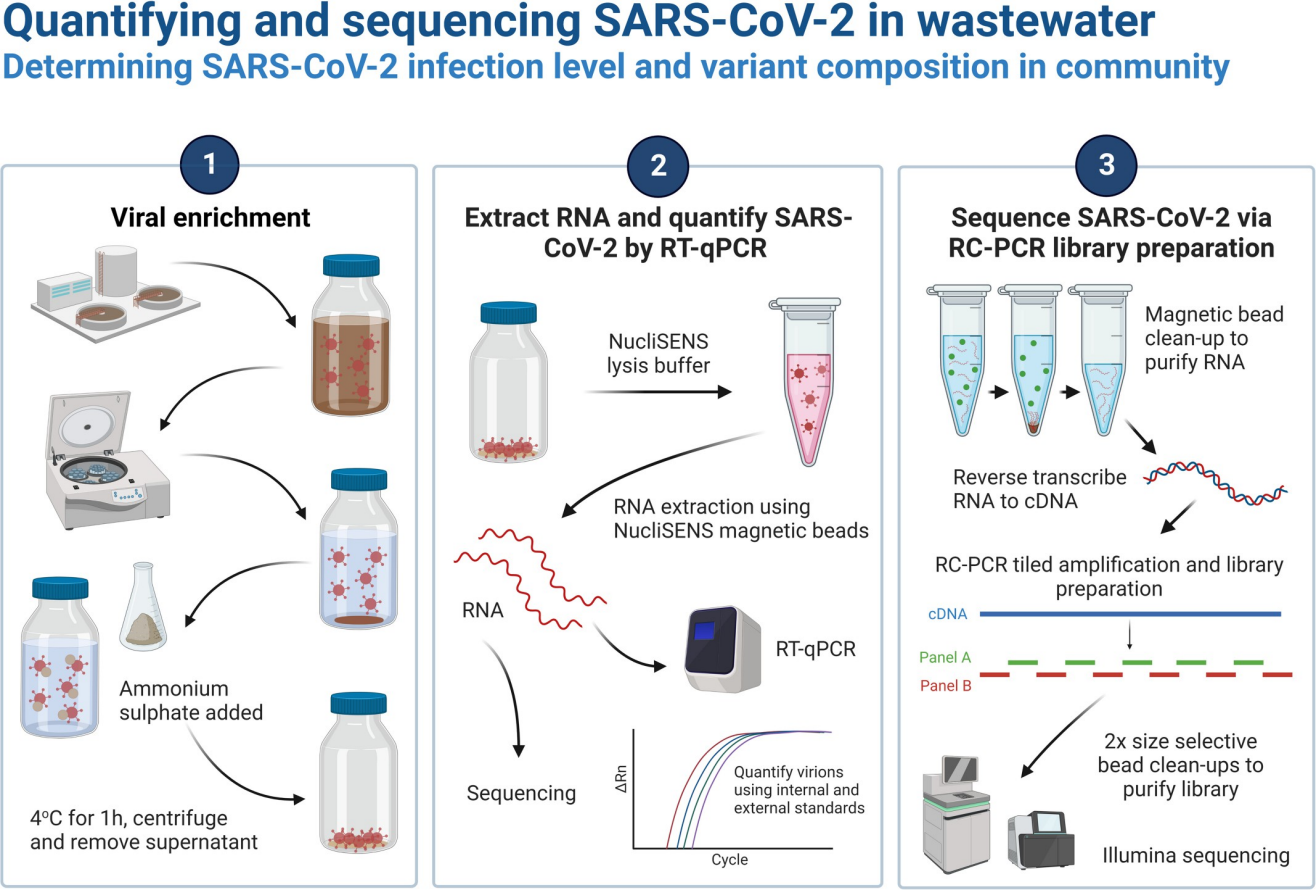
Overview of the protocol used for quantifying and sequencing SARS-CoV-2 in wastewater. Created with Biorender.com.

Here, we describe a protocol for SARS-CoV-2 quantification and sequencing in wastewater, from processing of raw wastewater samples to library preparation and quality control of the sequencing data (Fig 1). The library preparation protocol described here uses the EasySeq™ RC-PCR SARS CoV-2 (novel coronavirus) Whole Genome Sequencing Kit (NimaGen, The Netherlands). This kit uses reverse complement PCR (RC-PCR) to combine tiled amplification across the SARS-CoV-2 genome with the addition of sequencing adapter and Unique Dual Index (UDI) sequences for multiplexed sequencing on Illumina platforms, significantly reducing the hands-on time required for library preparation [33]. Furthermore, the present workflow performs viral concentration via rapid precipitation with ammonium sulphate, one of the techniques previously validated for enrichment of SARS-CoV-2 in wastewater samples [29]. This workflow has been used by the Environmental Monitoring for Health Protection (EMHP) wastewater monitoring programme in England [34], enabling RT-qPCR quantification and sequencing of SARS-CoV-2 in wastewater samples encompassing a population of approximately 40 million people (~70% of the population of England). This protocol also forms the basis of academic research investigating SARS-CoV-2 whole genome sequencing for wastewater-based epidemiology [26]. We present data from optimisation of the library preparation protocol, evaluation of sequencing protocol performance using synthetic SARS-CoV-2 RNA and typical sequencing results from its application to wastewater samples.

## Materials and methods

### RNA samples

Wastewater RNA samples used to generate sequencing data presented in this study were selected from those collected as part of the EMHP wastewater programme in England [34]. Wastewater samples used in demonstrating the impact of the RNA clean-up step (n = 74) were collected in June 2021. A separate set of samples used for presentation of detailed sequencing results (n = 77) were collected in January 2022. A sequencing library prepared with a set of 95 wastewater samples from March 2022 was used in the comparison of beads for post-PCR clean-up. Sequencing data from all 938 EMHP samples sequenced at the University of Exeter from March 2022 were used for demonstration of high-throughput wastewater surveillance using this protocol.

Synthetic SARS-CoV-2 RNA was obtained from Twist Biosciences (San Francisco, USA) and used to prepare variant mixtures and serial dilutions, including Control 15 Alpha (EPI_ISL_601443), Control 16 Beta (B.1.351; EPI_ISL_678597), Control 23 Delta (B.1.617.2; EPI_ISL_1544014) and Control 29 Delta (AY.2; EPI_ISL_2693246). Details of concentrations of each variant are provided in the table in S2 File.

### Wastewater processing and nucleic acid extraction and RT-qPCR

Details of the protocol for processing of wastewater samples, including sample clarification, viral enrichment and RNA extraction, and RT-qPCR SARS-CoV-2 quantification can be found in Walker et al. [35]. In brief, wastewater samples were clarified by centrifugation before viral enrichment by ammonium sulphate precipitation and nucleic acid extraction and purification using NucliSENS magnetic extraction reagents (bioMérieux, UK) on the Kingfisher Flex purification system (ThermoFisher, UK). RT-qPCR was carried out using the SARS-CoV-2 nucleocapsid gene N1 region as a target and a phi6 process control, both with single stranded RNA quantification standard dilutions, with the N1 region limit of detection of 0.4 gc/μL and limit of quantification of 4 gc/μL [35]. These steps were carried out by the Environment Agency (Exeter, UK).

### Library preparation by reverse complement PCR

The protocol described in this peer-reviewed article for SARS-CoV-2 amplicon library preparation and sequencing in wastewater RNA samples is published on protocols.io (dx.doi.org/10.17504/protocols.io.81wgb7bx3vpk/v3) and is included for printing as S1 File with this article. In brief, wastewater nucleic acid samples are purified by 1.8x magnetic bead clean-up using Mag-Bind® TotalPure NGS beads (Omega Bio-tek) before cDNA is synthesised using the LunaScript® RT SuperMix Kit (New England Biolabs, UK). This protocol then utilises the EasySeq™ RC-PCR SARS CoV-2 (novel coronavirus) Whole Genome Sequencing Kit (NimaGen, The Netherlands) for library preparation, which generates SARS-CoV-2 amplicons with sequencing adapters and indexes in one step. Wastewater samples for which sequencing data is presented here were processed using the v4.01 probe set from NimaGen. Finally, libraries are purified by two consecutive 0.6x magnetic bead clean-ups with Mag-Bind® beads, to remove primers and PCR artifacts, before being sequenced. Sequencing of samples included in this article was carried out on a NovaSeq 6000 2 × 150 bp SP flowcell (Illumina, UK).

### Sequencing data processing and QC

Raw Illumina sequences were analysed using the Illumina workflow of the ncov2019-artic-nf pipeline (https://github.com/connor-lab/ncov2019-artic-nf), and a BED file supplied by Nimagen containing the primer v4.01 locations. Briefly, reads were trimmed using Trim Galore! V0.6.5 (https://www.bioinformatics.babraham.ac.uk/projects/trim_galore/) and subsequently mapped to the SARS-CoV-2 reference genome [11] (GenBank: MN908947.3) using BWA v0.7.17 [36] and sorted using samtools v1.10 [37]. Primer sequences were trimmed (masked) using iVAR v1.3 [38] and only mapped reads that contained primer sequences were included in subsequent analyses (—allowNoprimer false).

For each sample, the percentage of quality-trimmed reads that mapped to the SARS-CoV-2 genome after primer-trimming (as a percentage of total number of raw reads generated), percentage of the genome covered, and mean coverage depth were calculated. Results obtained with the different post-PCR clean-up methods (Mag-Bind® TotalPure NGS or NimaGen AmpliCleanTM beads), and standard versus additional RNA clean-up steps, were compared and visualised as scatterplots using custom scripts in R (S3 File).

Medium coverage depths for each of the 154 amplicons were determined for each sample using mosdepth v0.2.6 (using fast-mode and the following thresholds: 0, 1, 10, 50, 100, 500, 1000, 5000, 10000, 50000) and heatmaps for the synthetic SARS-CoV-2 lineage mixtures and wastewater samples were generated in R using custom scripts (S3 File).

Relative SARS-CoV-2 lineage abundances were estimated using Freyja [27] (v1.3.1; https://github.com/andersen-lab/Freyja; curated lineage file and UShER global phylogenetic tree downloaded on 31st Dec 2021). Expected and measured abundances as well as percentage genome coverage were plotted using custom scripts in R (S3 File).

## Results

### Wastewater RNA clean-up improved library quality and genome coverage

**Fig 2:**
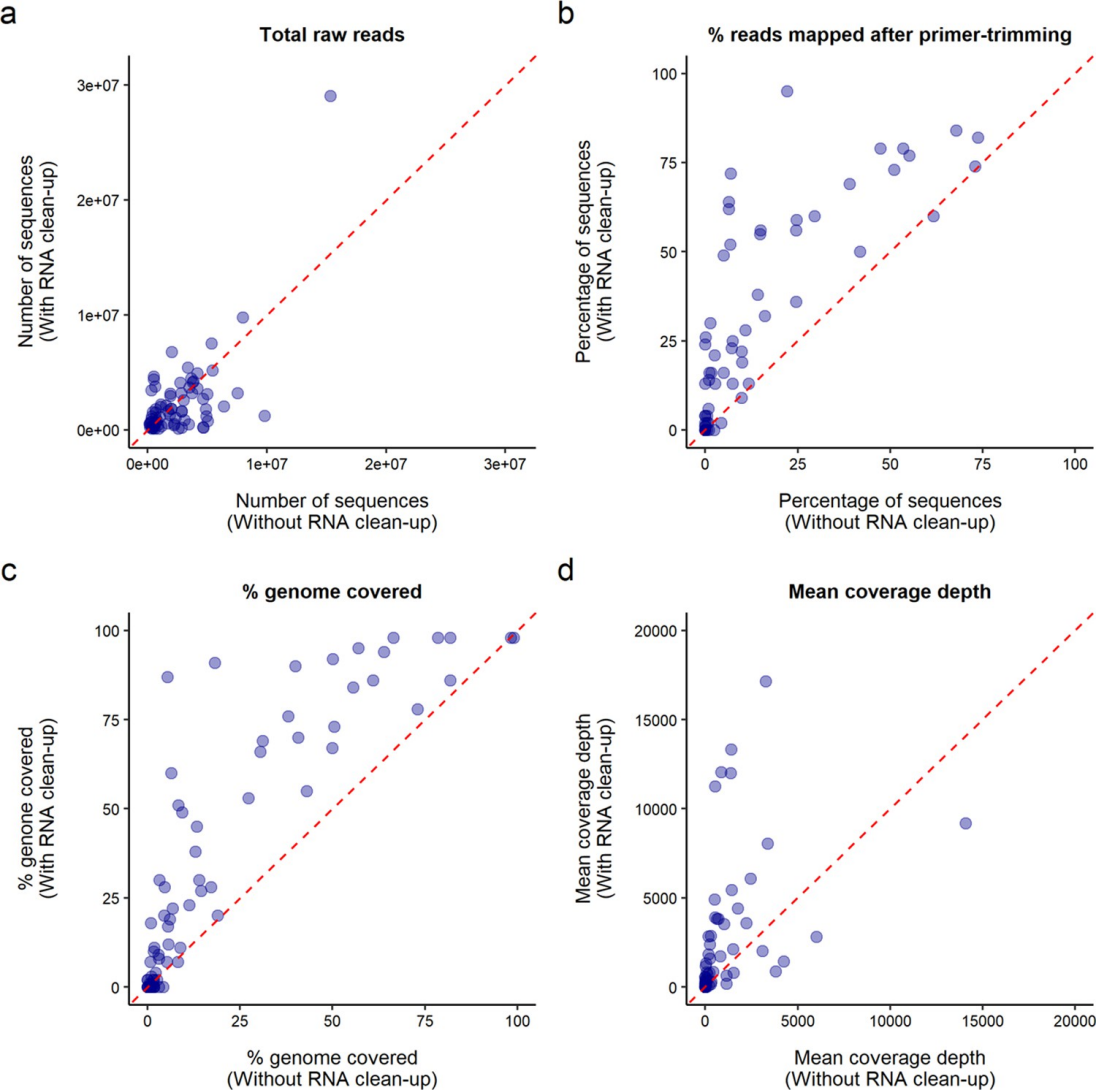
RNA clean-up resulted in improved library preparation and sequencing. Sequencing and alignment data, including total number of raw reads (a), percentage of raw reads mapping to SARS-CoV-2 genome after primer-trimming (b), percentage coverage breadth (c) and mean coverage depth (d), for each sample sequenced with or without the RNA clean-up step. Red line delineates x = y.

The initial step in the library preparation protocol described here involves purification of extracted RNA using Mag-Bind® TotalPure NGS (Omega Bio-tek) magnetic beads. This step was found to significantly improve the quality of sequencing libraries during optimisation of the protocol. To demonstrate this, a set of 74 wastewater samples, collected in June 2021 from sewage network sites across England, were sequenced in parallel through the protocol with and without the RNA clean-up step. The number of raw reads obtained for each sample was not skewed towards either of the two parallel libraries, demonstrating effective normalisation of these libraries during sequencing (Fig 2A). When samples were subjected to RNA clean-up, a higher percentage of sequencing reads mapped to the SARS-CoV-2 genome after primer-trimming (Fig 2B), indicating that SARS-CoV-2 cDNA recovery and PCR amplification were more efficient when including this step. Improved genome coverage and coverage depths were also obtained when the RNA clean-up was performed (Fig 2C and 2D). This improvement in library preparation is likely the result of removing inhibitors to reverse transcription and PCR through RNA clean-up. These results show the importance of this initial processing step to improving wastewater sequencing.

### Implementation of two post-PCR clean-up steps improved library quality

**Fig 3:**
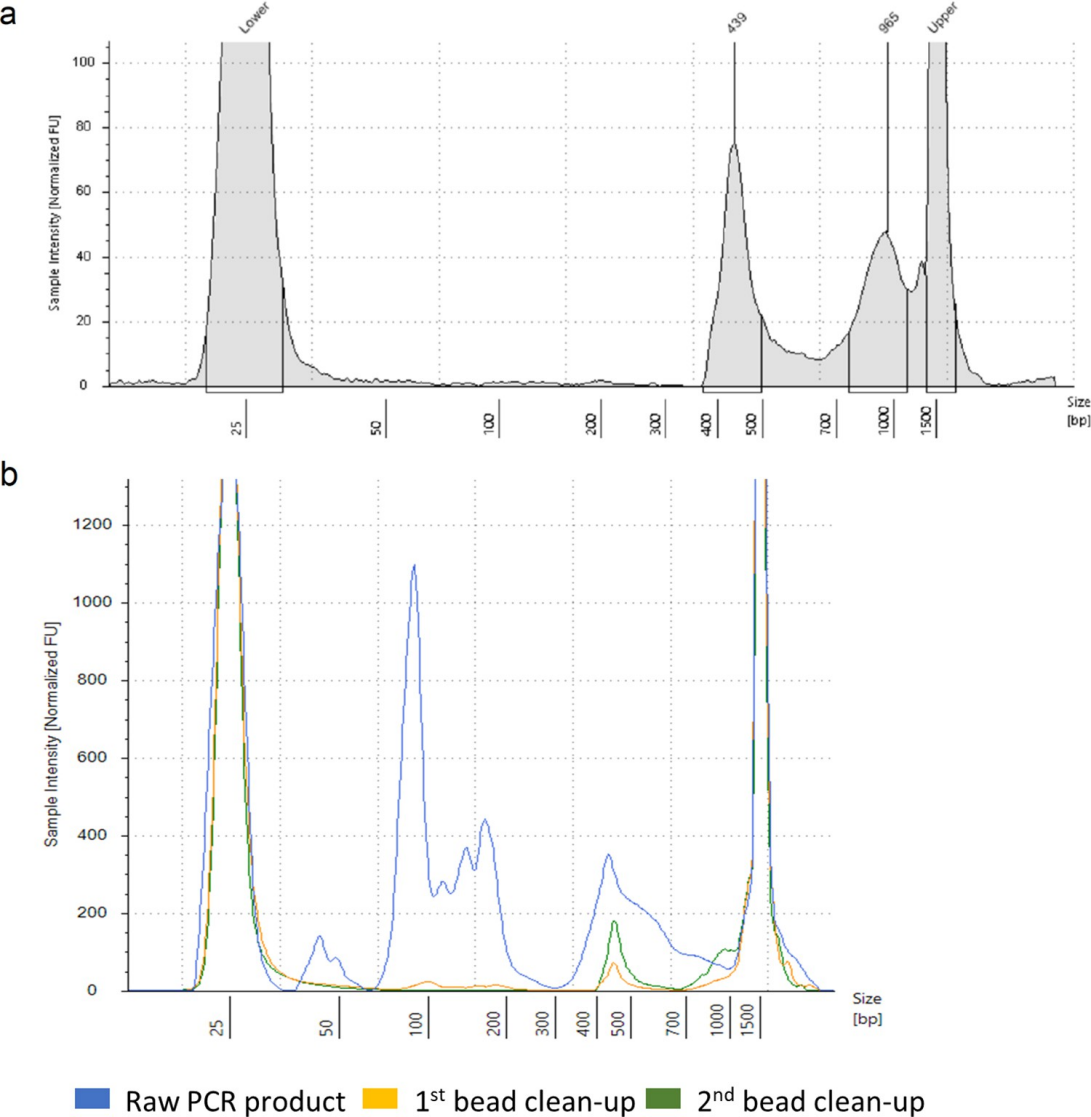
Library quality control using fragment size analysis demonstrated importance of two post-PCR clean-up steps. (a) Typical size profile from Agilent D1000 ScreenTape assay of a wastewater sequencing library following post-PCR clean-up and (b) overlayed electropherograms showing size profiles of raw PCR products and after 2 consecutive bead clean-up steps.

Quality control (QC) of amplicon libraries generated with this protocol is crucial prior to sequencing. The main method of routine library QC is fragment size analysis using the Agilent D1000 ScreenTape assay on the 4200 TapeStation system (Agilent, UK). Similar fragment size analysis could be carried out using an Bioanalyzer system (Agilent, UK), or gel electrophoresis if the former technologies are unavailable. This QC assay captured the success of the RC-PCR reaction in amplifying the desired SARS-CoV-2 amplicon library of around 435 bp, as well as the efficacy of the size selection in purifying these amplicons. A typical size profile of the libraries following post-PCR purification is provided in Fig 3A. The importance of the post-PCR magnetic bead clean-up procedure is demonstrated by fragment size analysis of a pooled library before, between and after two consecutive 0.6x bead clean-ups using Mag-Bind® TotalPure NGS beads (Fig 3B). Before the clean-up, large amounts of small fragments, including primer dimers and PCR artifacts, dominate the pooled products (Fig 3B). Significant amounts of these small fragments remain after the first clean-up, while the second clean-up reduces this to trace amounts (Fig 3B). If a significant level of small fragments (relative to the desired amplicon peak) is identified through library QC, an additional size-selective bead clean-up can be performed to further purify the library.

**Fig 4:**
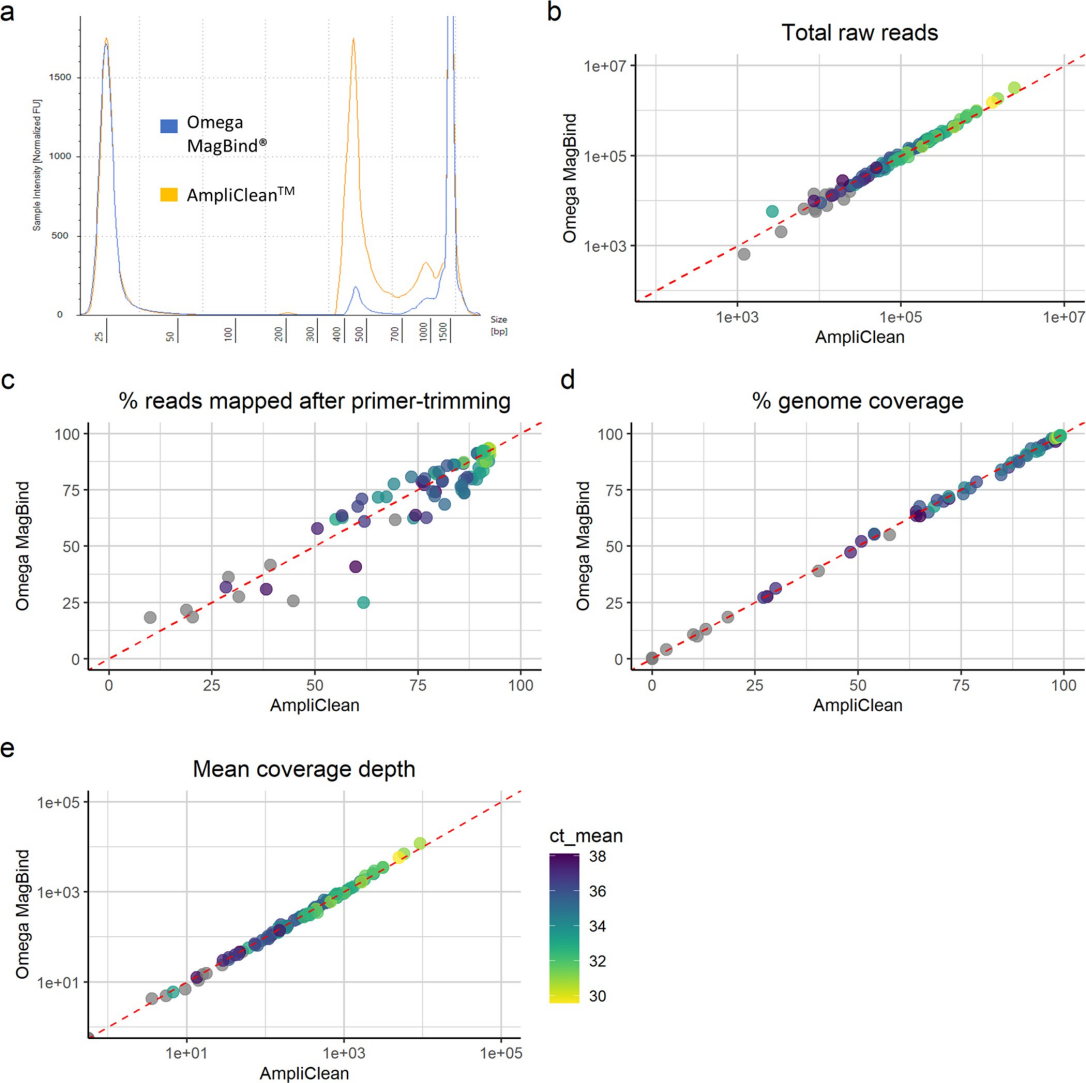
Post-PCR clean-up was effective with either Mag-Bind® TotalPure NGS or NimaGen AmpliClean^TM^ beads. (a) Overlayed fragment size profiles of sequencing libraries purified in parallel with each bead type. Sequencing and alignment data, including total number of raw reads (a), percentage of raw reads mapping to SARS-CoV-2 genome after primer-trimming (b), percentage coverage breadth (c) and mean coverage depth (d), for each sample following purification with each bead type, with the colour of points representing the mean Ct value from SARS-CoV-2 RT-qPCR of the sample.

During the initial optimisation of the protocol, post-PCR purification by 0.6x clean-up with Mag-Bind® beads was found to be more effective than 0.85x clean-up with the beads supplied with the NimaGen EasySeq™ kits and detailed in the NimaGen protocol. However, NimaGen have since optimised the composition of the AmpliClean^TM^ beads supplied, and comparison of these with Mag-Bind® beads has shown equivalent performance for wastewater SARS-CoV-2 library preparation (Fig 4). The same pooled PCR products were purified in parallel by two consecutive bead clean-up steps using 0.6x Mag-Bind® beads, following the protocol described here, and 0.85x AmpliClean^TM^ beads, as detailed in the manufacturers protocol. Fragment size QC of the resulting libraries demonstrated effective purification of the desired SARS-CoV-2 amplicons by both bead types, while the yield of amplicons was significantly higher in libraries purified with AmpliClean^TM^ beads (Fig 4A). Sequencing of these libraries led to a similar number of raw reads acquired for each sample (Fig 4B). Furthermore, alignment to the SARS-CoV-2 genome resulted in a similar percentage of raw reads mapped after primer-trimming across all samples (Fig 4C), indicating that both bead types show similar performance in removing PCR artifacts. This is further supported by the equivalent percentage genome coverage and mean coverage depth obtained for all samples (Fig 4D and 4E). Overall, these findings support the efficacy of using the AmpliClean^TM^ beads in place of the Mag-Bind® beads in the protocol presented here.

### Performance evaluation using synthetic SARS-CoV-2 RNA samples

**Fig 5:**
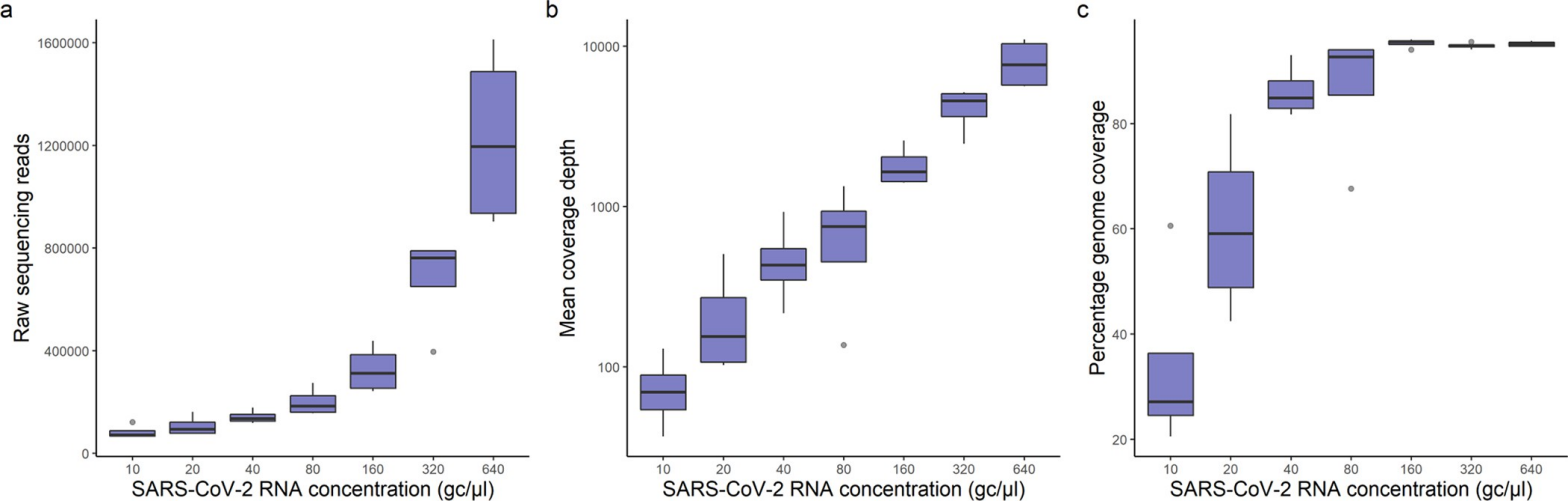
Sequencing metrics from serial dilutions of synthetic SARS-CoV-2 RNA. Boxplots showing total number of raw reads (a), mean coverage depth (b) and percentage coverage breadth (c) obtained at a range of starting RNA concentrations in genome copies/μl (gc/μl).

**Fig 6:**
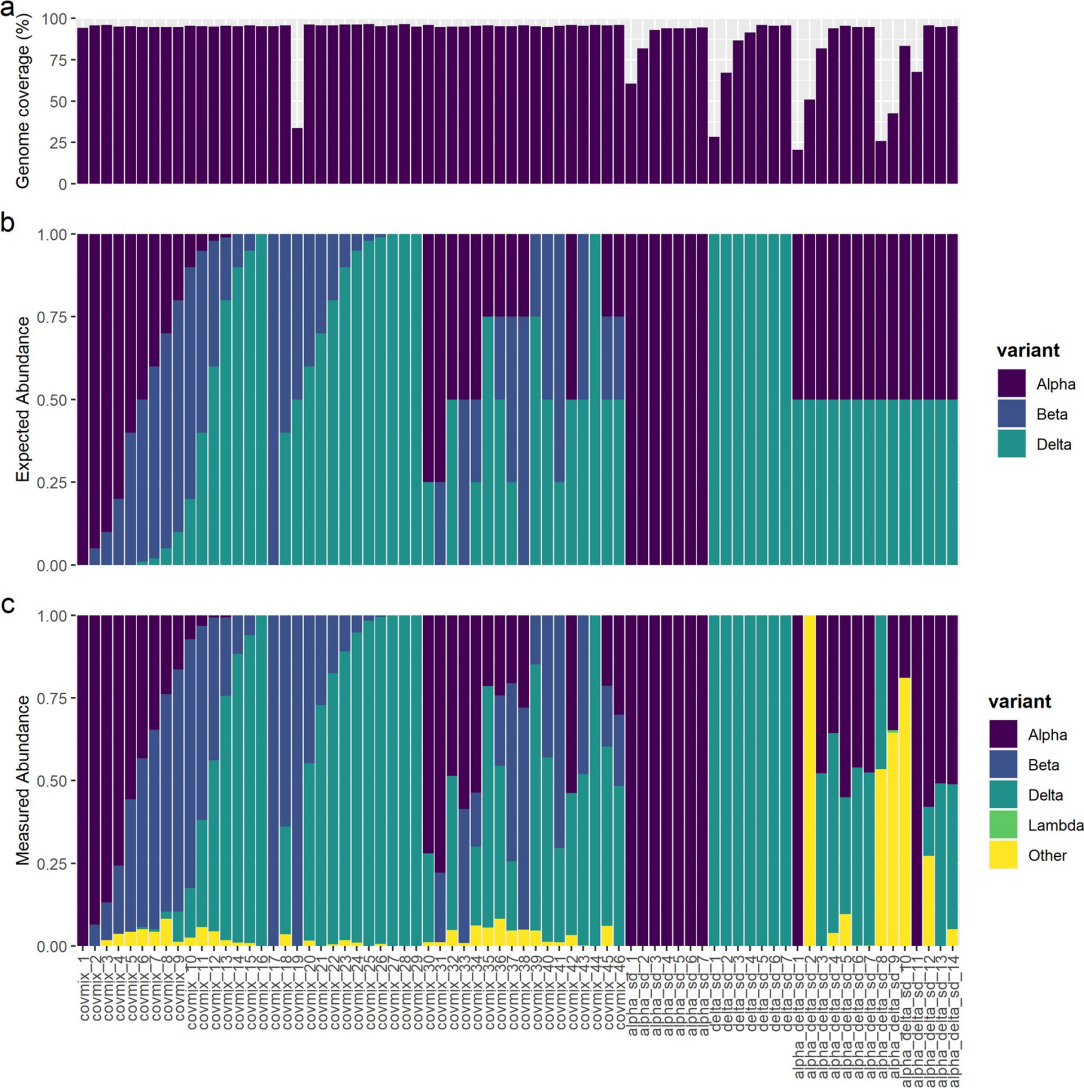
Wastewater sequencing protocol can successfully decipher synthetic SARS-CoV-2 variant mixtures. (a) Percentage genome coverage obtained for each synthetic SARS-CoV-2 RNA sample. (b) Expected abundances based on the concentration of each synthetic variant in each sample. (c) The measured abundance of each variant from analysis of sequencing data using Freyja.

The library preparation protocol was validated using synthetic SARS-CoV-2 RNA. To investigate the efficacy of the protocol in sequencing samples with a range of SARS-CoV-2 concentrations, a two-fold dilution series was prepared from 640 to 10 genome copies (gc)/μl using the variants Alpha and Delta (B.1.617.2), as well as an equal parts Alpha/Delta mixture (S2 File). The number of raw sequencing reads per sample was found to increase in line with SARS-CoV-2 RNA concentration (Fig 5A), which is expected when post-PCR pooling is carried out without normalisation based on template concentration. Consequently, mean sequencing depth was also found to decrease at lower starting SARS-CoV-2 RNA concentrations (Fig 5B). All samples with concentrations ≥160 gc/μl had a coverage breadth >94% (Fig 5C and Fig 6A). This is approaching the maximum achievable coverage considering the synthetic SARS-CoV-2 RNA was synthesised in six non-overlapping 5kb fragments, causing amplicons bridging these gaps to drop out. Coverage breadth was found to decrease for samples with SARS-CoV-2 RNA concentration below 160 gc/μl, although partial genome coverage (20–60%) was still achieved at 10 gc/μl (Fig 5C and Fig 6A).

SARS-CoV-2 lineage calling using Freyja [27] was able to accurately determine the variant of single-strain serial dilutions at all concentrations (Fig 6B/6C). Using sequencing data from the serial dilutions of equal mixtures of Alpha and Delta, variant calling of replicate 1 (“alpha_delta_sd_1–7”) successfully determined similar abundances of both strains down to a concentration of 40 gc/μl (Fig 6B/6C). However, for replicate 2 (“alpha_delta_sd_8–14”), significant abundances of both Alpha and Delta were only determined for samples above 160 gc/μl (Fig 6B/6C), despite coverage breadth of 67% and 83% for samples at 80 gc/μl (“alpha_delta_sd_11”) and 40 gc/μl (“alpha_delta_sd_10”), respectively (Fig 6A). These results highlight the difficulty in deciphering lineage abundances when multiple variants are present in samples, which is vulnerable to any decrease in coverage breadth causing the absence of data for lineage defining mutations.

To further assess the efficacy of the library preparation protocol and QC pipeline, including differentiating between SARS-CoV-2 variants using Freyja, a range of proportions of the variants Alpha, Beta, Delta and Delta AY.2 were used to prepare 46 samples (“covmix1-46”) at a concentration of 200 gc /μl (S2 File). Genome coverage breadth of >94% and mean depth of 5,000–15,000x was obtained for all variant mixtures apart from “covmix_19”, which had a breadth of 33% and depth of 74x due to an undetermined issue with this sample (Fig 6A). The relative abundance of variants in these samples determined by Freyja showed high similarity to the expected proportions based on composition of the sample (Fig 6B and 6C). This provides evidence that the library preparation protocol here along with variant calling with Freyja can quantitatively determine the relative frequencies of SARS-CoV-2 variants in RNA samples, provided that the breadth of genome coverage is sufficient to cover lineage defining SNPs.

Overall, these results demonstrate the efficacy of this protocol for sequencing of SARS-CoV-2, including mixtures of variants expected to be present in wastewater samples. We recommend similar performance evaluation, using synthetic SARS-CoV-2 RNA or clinical samples as positive controls, in any laboratory implementing this protocol.

### Sequencing results for wastewater samples

**Fig 7:**
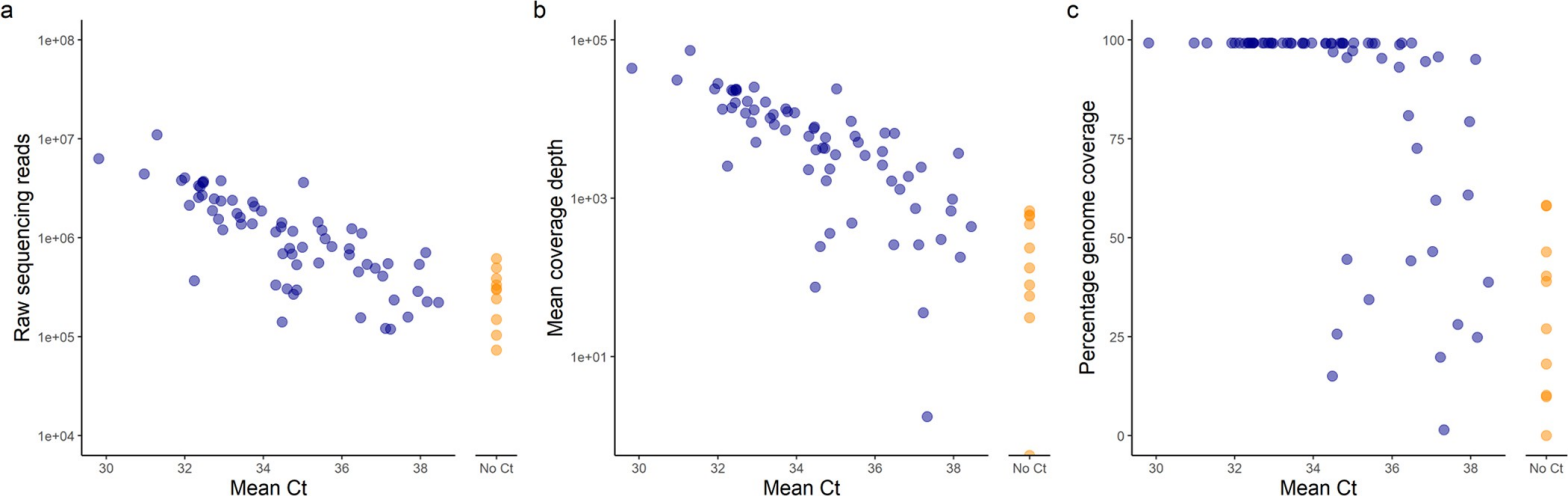
Sequencing results from a set of 77 wastewater samples. Scatterplots demonstrating how sequencing and alignment data, including total number of raw reads (a), mean coverage depth (b) and percentage coverage breadth (c), vary across samples with a range of Ct values from SARS-CoV-2 RT-qPCR. Colour of points represents whether a Ct value was determined (blue) or not determined (orange).

**Fig 8:**
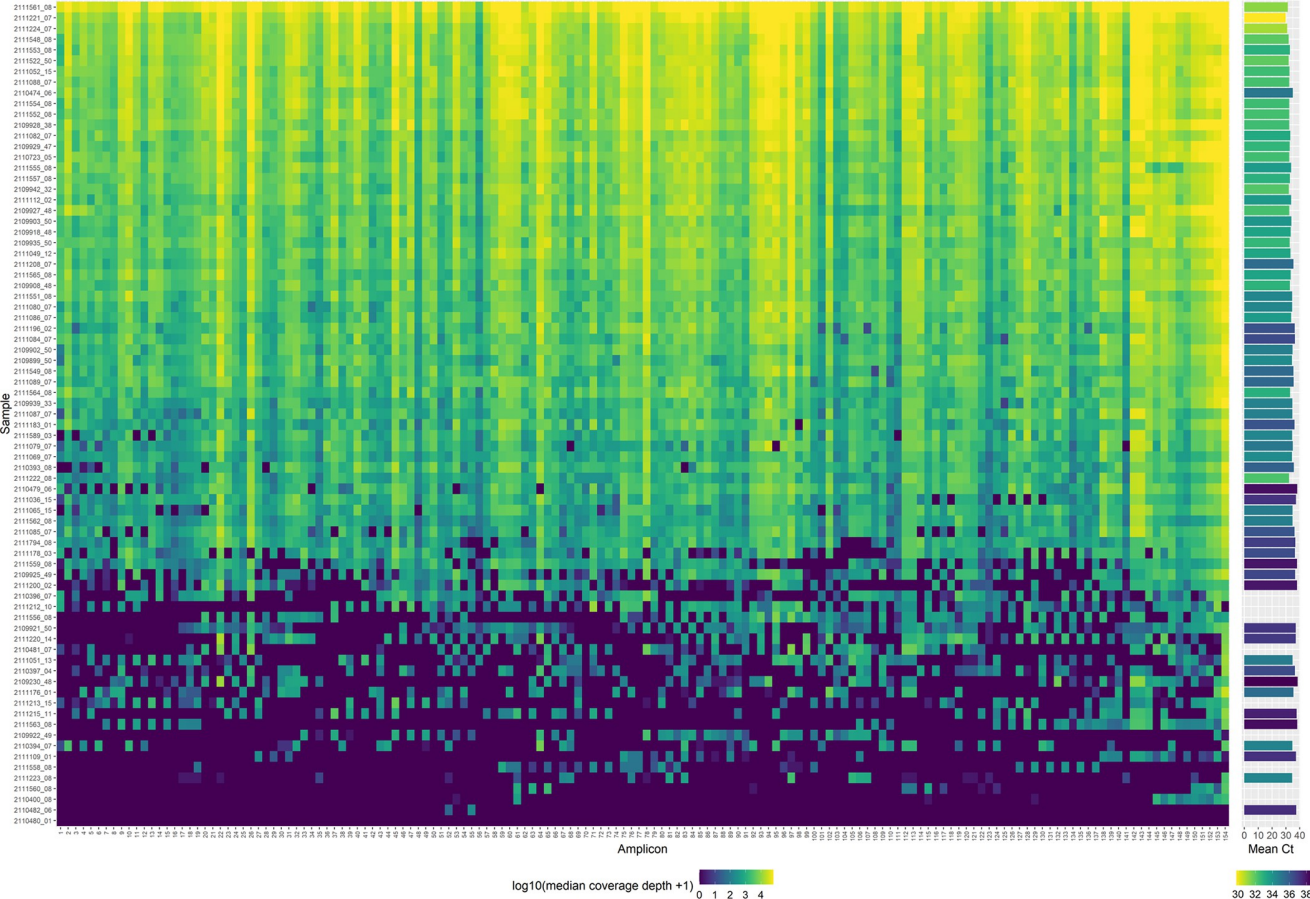
Coverage depth across the SARS-CoV-2 genome. Heatmap visualising median coverage depth across each amplicon (left) for 77 sequenced wastewater samples, with the associated SARS-CoV-2 Ct value for each sample (right). Ct value of 0 refers to samples for which RT-qPCR failed to identify SARS-CoV-2.

Sequencing results generated using this protocol are presented here for 77 wastewater samples collected on 18^th^ January 2022 from sewer network sites across England. The resulting libraries were sequenced on a NovaSeq SP flowcell, with an average of 1.5 million 2x150 bp paired-end sequencing reads generated per sample. As expected with equal pooling of PCR products from all samples, regardless of estimated SARS-CoV-2 concentration, samples with lower Ct values generated more sequencing reads and had higher mean coverage depth (Fig 7A and 7B). Over 99% genome coverage was obtained for all samples with Ct<34 (~28 gc/μl in RNA before clean-up; Fig 7C). Furthermore. genome coverage >95% was obtained for 64% of samples, including those with Ct values up to 38 (~1 gc/μl in RNA before clean-up; Fig 8C), and partial genome coverage was achieved for all but one sample. Variant calling using Freyja resulted in over 99.5% abundance of Omicron for all samples with genome coverage, corresponding with the dominance of this variant in the population at the time of sampling [39]. These results demonstrate the high sensitivity of the protocol presented here.

Considering Ct values only represent quantification of one genomic region of the sample, corresponding to amplicon 148, we found that high genome coverage breadth and depth can be obtained despite low SARS-CoV-2 concentration indicated by RT-qPCR analysis (Fig 7B and 7C). Furthermore, 9 of the 10 samples for which no SARS-CoV-2 could be detected by RT-qPCR had a mean coverage depth of between 170-1520x and a coverage breadth of 10–60% (Fig 7B and 7C), indicating that fragmented SARS-CoV-2 RNA was present in these samples. The failure to detect SARS-CoV-2 by RT-qPCR in these samples could also be caused by the presence of inhibitors in samples prior to RT-qPCR, especially considering the lenient positive control recovery threshold used [35] and the high levels of RT-qPCR inhibition recently identified in this assay [40]. Such inhibitors could potentially have been removed during bead purification of RNA before cDNA synthesis. These findings further highlight the sensitivity offered by whole-genome amplicon sequencing for SARS-CoV-2 surveillance when compared to RT-qPCR analysis.

Although the percentage genome coverage and mean coverage depth give a good indication of the success of sequencing, assessing the coverage depth across each amplicon provides a more detailed level of quality control (Fig 8). Plotting a heatmap of median coverage depth across each amplicon provides a visual assessment of the success of each of the PCR reactions, with alternating amplicons from the A and B probe sets, as well as evidence of any amplicon dropouts across samples which may be caused by the presence of mutations in the primer regions. In the case of the sequencing data presented here, even coverage of amplicons was achieved for most samples (Fig 8). Samples with lower coverage breadth, often associated with low SARS-CoV-2 template concentration characterised by high or undetermined Ct values, frequently display dropout of amplicons from one of the PCR probe sets across different regions (Fig 8). This is likely caused by the segregation of low copy number SARS-CoV-2 cDNA fragments between the A and B reactions during PCR set up.

### Wastewater sequencing from national surveillance programme

**Fig 9:**
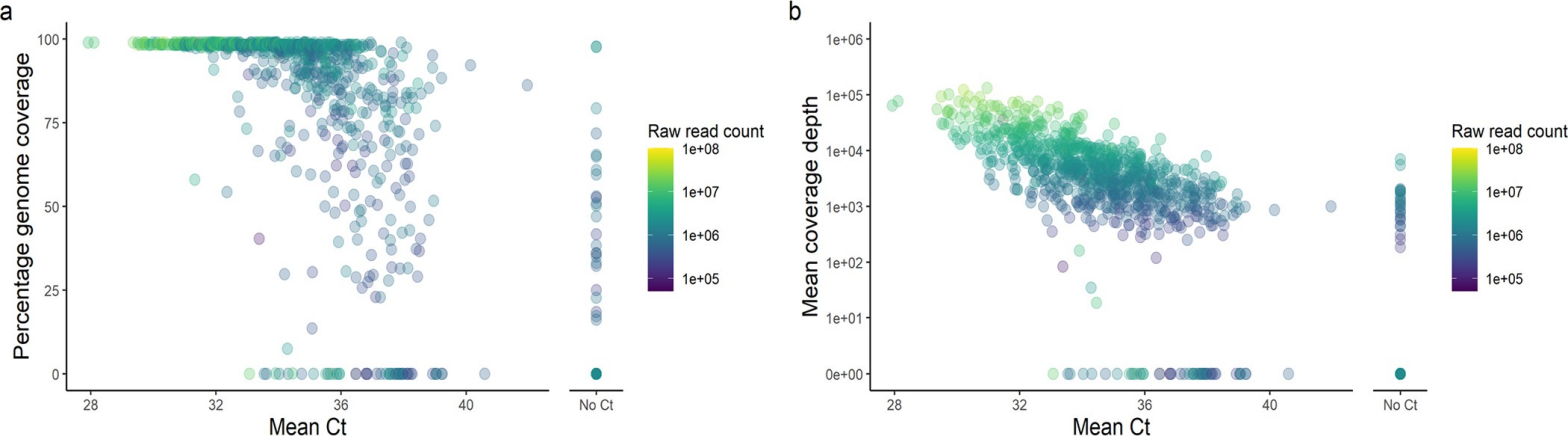
Results from high-throughput wastewater SARS-CoV-2 sequencing. Scatterplots of percentage coverage breadth (a) and mean coverage depth (b) against Ct values from SARS-CoV-2 RT-qPCR. Colour of points represents the number of raw sequencing reads obtained for each sample.

The present protocol has been used for high-throughput sequencing of wastewater samples from across England as part of the Environmental Monitoring for Health Protection (EMHP) wastewater monitoring programme [34]. To demonstrate the reliable sequencing results generated by this high-throughput monitoring, sequencing metrics are presented here for 938 untreated influent samples taken from network sites (manholes) across England in March 2022, sequenced on NovaSeq SP flowcells at the University of Exeter (Fig 9). Genome coverage above 80% was obtained for 87% of samples with Ct<37, while genome coverage between 16–98% was obtained for 28% of samples for which no Ct value was obtained by RT-qPCR (Fig 9A). These findings demonstrate the sensitivity of the protocol to acquire broad coverage of the SARS-CoV-2 genome in samples with low template concentration. SARS-COV-2 variant definitions include specific mutations distributed across the genome (https://github.com/phe-genomics/variant_definitions), meaning the high genome coverage observed is essential for detecting the presence of mutation profiles to support the assignment of variant(s) to samples. Furthermore, with a raw sequencing read depth of >150,000, a mean coverage depth of >200x was obtained for most of the samples (Fig 9B), providing ample coverage for calling polymorphisms from lineages of low abundance.

## Discussion

### Viral enrichment in wastewater

Although some SARS-CoV-2 wastewater monitoring studies have utilised primary settled sewage samples [10], influent wastewater has more often been used as the initial sample [23, 24, 41], which was recently shown to yield improved sequencing results [42]. Wastewater samples are typically clarified by filtration or centrifugation to remove large particles, before a viral enrichment step is employed to increase SARS-CoV-2 concentration. This is achieved by ultrafiltration in most studies [8, 20, 21, 23–25, 41, 42]. However, cheaper alternatives such as precipitation with polyethylene glycol (PEG), ammonium sulphate or skimmed milk have been demonstrated to be similarly effective for SARS-CoV-2 recovery, as well as being more robust to changes in wastewater variables, such as turbidity, and between sample collection sites, compared to ultrafiltration [29, 30]. During extraction of wastewater nucleic acid samples sequenced using this protocol, viral enrichment was carried out by precipitation with ammonium sulphate (Fig 1) [35]. This method circumvented the issue of filter blockages encountered during enrichment by centrifuge-based ultrafiltration [35]. Furthermore, ammonium sulphate precipitation increased the overall processing efficiency due to the 1-hour incubation period required, compared to overnight incubation required for PEG precipitation [29, 35].

### RC-PCR library preparation

**Fig 10:**
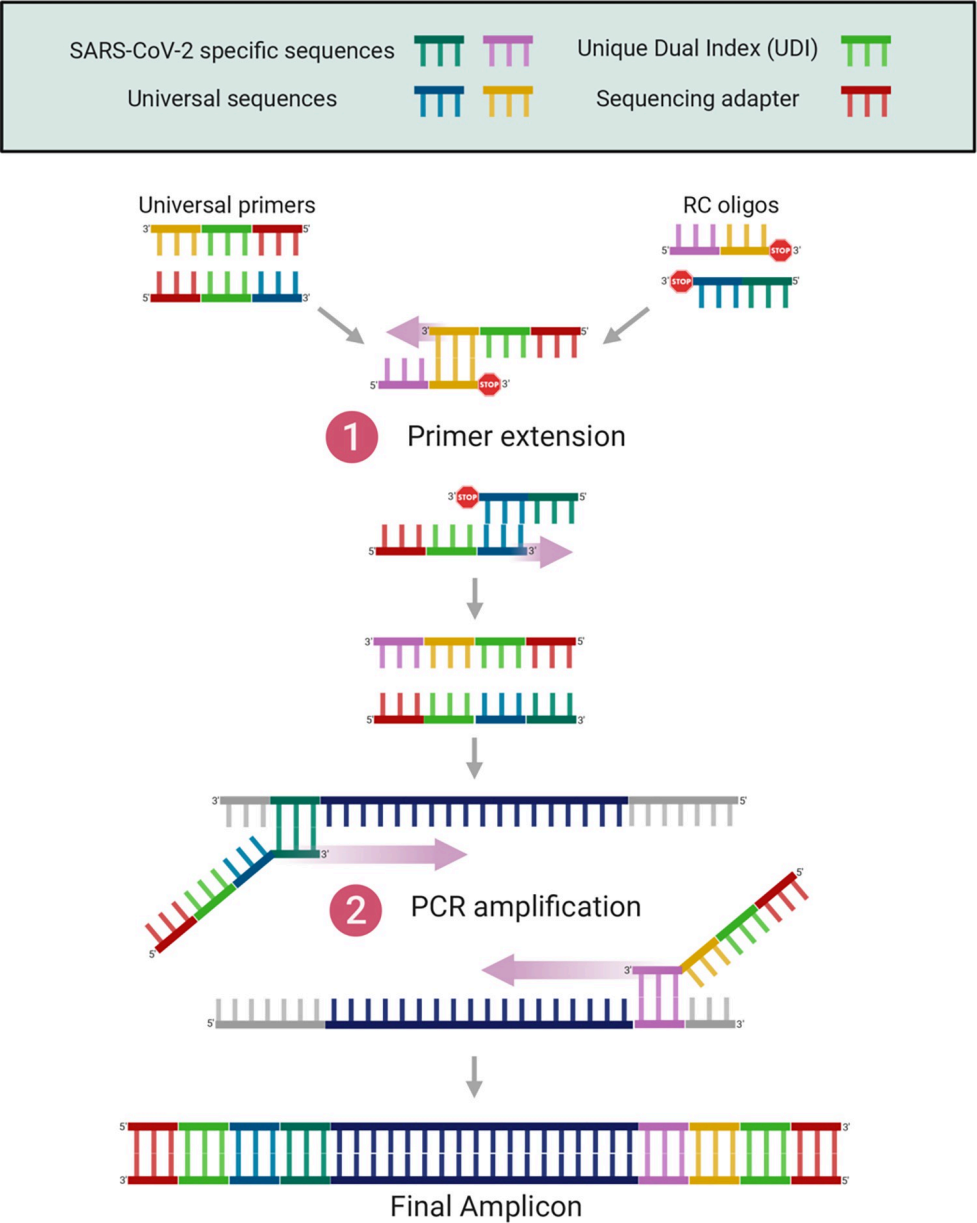
Schematic describing the reverse complement PCR method. Created with Biorender.com, based on adaptation from Coolen et al. (2021).

The NimaGen EasySeq™ RC-PCR SARS CoV-2 (novel coronavirus) Whole Genome Sequencing Kit used in this protocol utilises RC-PCR to integrate PCR amplification and multiplexed library preparation for Illumina sequencing [33]. The first stage of RC-PCR consists of the formation of target specific probes from universal primers, containing sequencing adapters and Unique Dual Index (UDI) sequences, and RC oligos, which contain the reverse compliment of the SARS-CoV-2 specific sequences (Fig 10). Universal primers and RC oligos bind via complementary universal sequences, upon which the SARS-CoV-2-specific sequences are synthesised to complete the SARS-CoV-2 specific primers (Fig 10). The RC probe is blocked from extension at the 3’ end, ensuring only one orientation of each SARS-CoV-2-specific primer is assembled. Following this, normal PCR cycles generate tiled amplicons containing Illumina sequencing adapters at each end (Fig 10). Each well of the “IDX” PCR plate contains universal primers with distinct UDI sequences, resulting in unique barcodes being added to the amplicons for each sample.

Overall, one-step PCR and adapter incorporation significantly reduces hands-on time over other amplicon library preparation methods. Indeed, RC-PCR preparation takes less than 30 min for one 96 well plate, and hands-on time for the protocol presented here is less than 4 hours, which is significantly less than other protocols such as ARTIC and Midnight [43]. The RC-PCR thermal cycler protocol takes 6–7 hours and is therefore suited to an overnight step. Considering that post-PCR clean-up and library QC can be completed in 2 hours, it is feasible to process samples from receipt to loading onto the sequencer in less than 24 hours using this protocol. Along with the ~24–26 h required for a 2x150 bp run on Illumina platforms and sequence data analysis, this method is therefore feasible to complete from RNA sample to analysed sequencing data in three working days. Furthermore, this library preparation protocol is amenable to high-throughput sample processing, with 768 dual-separate barcodes currently utilised by the NimaGen 96-well kits and up to 1,536 in the 384-well format.

The primer scheme NimaGen EasySeq™ generates amplicons with a mean insert size of 226 bp, which are shorter than other popular SARS-CoV-2 amplicon schemes such as those in the ARTIC (~400bp) [32] and the Midnight (~1200bp) [31] protocols. Primer pairs from schemes with shorter inserts are more likely to bind contiguously on shorter DNA fragments, and are therefore more robust to RNA degradation in the sample [32], while longer amplicons reduce variation in coverage depth across the genome, achieving better genome coverage with less sequencing depth [31]. It has been proposed that shorter amplicons, such as those generated in the NimaGen EasySeq™ kit, could therefore be better suited to wastewater samples which may be more prone to degradation, with a previous study finding improved coverage breadth for high Ct wastewater samples using the ARTIC compared to the Midnight protocol [42]. Alongside the aforementioned benefits to efficiency and throughput capacity, the NimaGen EasySeq™ kit was chosen for SARS-CoV-2 wastewater library preparation.

### Controls

Due to the high-throughput nature of regular wastewater sequencing using this protocol, and the low SARS-CoV-2 concentration commonly associated with wastewater RNA samples, pooling of amplicon libraries was carried out routinely without normalisation. However, when positive control material from clinical samples or synthetic SARS-CoV-2 RNA, which typically contain high SARS-CoV-2 concentrations, is used alongside wastewater samples, post-PCR dilution and/or adjustment of pooling volumes is recommended to prevent controls dominating the obtained sequencing reads. For smaller numbers of samples, or when samples of atypically high SARS-CoV-2 concentration are processed (i.e. <Ct 28), normalisation during pooling of PCR products based on Ct values from SARS-CoV-2 RT-qPCR would improve balancing of read depth across samples.

The inclusion of negative controls is important to ensure no contamination has been introduced into the PCR master mix from previous SARS-CoV-2 PCR runs. To avoid introduction of amplicon contamination, PCR master mix should be prepared in a PCR-cabinet, and post-PCR purification and library QC should be performed in a separate area, or preferably room, within the lab and with different equipment (i.e., pipettes/centrifuges etc.). Sequencing reads from negative controls are routinely found to align to the SARS-CoV-2 genome due to the presence of PCR artifacts, the vast majority of which should be filtered out after primer-trimming. However, low levels (<0.1%) of reads from negative controls are routinely found to align to the SARS-CoV-2 genome after primer-trimming, likely due to contamination from previous amplicons with the same barcodes being introduced during post-PCR purification. The use of too many negative controls in pooled libraries may introduce higher levels of PCR artifacts into the final pool, due to high concentrations of these in PCR reactions containing no template, which may reduce the depth of sequencing for wastewater samples in these pools. We therefore recommend that a maximum of two negative controls are included in each 96-well plate.

### Sensitivity

The presence of PCR inhibitors in wastewater is widely recognised as one of the challenges of wastewater-based epidemiology [1, 44], as was recently shown in a study comparing SARS-COV-2 RT-qPCR assays in wastewater [40]. The additional RNA bead clean-up step included in this protocol led to dramatic improvements in library quality and SARS-CoV-2 genome coverage. Other SARS-CoV-2 wastewater sequencing protocols have also included additional RNA purification after extraction using the Zymo Research OneStep PCR Inhibitor Removal Kit [25, 45], although evidence for the impact of this step on sequencing results was not provided. Our findings indicate that additional RNA purification should be considered in the development of amplicon-based wastewater sequencing protocols.

We demonstrate that SARS-CoV-2 can be regularly detected through whole-genome sequencing in RT-qPCR-negative wastewater samples, often yielding sufficient genome coverage breadth and depth for variant assignment. This highlights the enhanced sensitivity of targeting amplicons across the genome compared to the short regions targeted by RT-qPCR, which is particularly effective for wastewater monitoring in which samples are expected to contain more degraded viral RNA than clinical samples obtained from nasopharyngeal swabs. Indeed, whole-genome sequencing has been proposed as a highly sensitive alternative diagnostic tool for SARS-CoV-2 detection in clinical samples [32, 46], although higher costs and longer turnaround times may limit its usage. In the case of this protocol, amplicon sequencing offers increased sensitivity for SARS-CoV-2 detection to complement the quantitative surveillance provided by RT-qPCR analysis.

## Conclusions

The protocol described here provides an optimised and efficient method for sequencing of viruses from influent wastewater, utilising reverse complement PCR for amplicon generation and library preparation in one step. This increases efficiency and reduces hands-on time compared to other protocols. We demonstrated the sensitivity of this protocol to provide broad coverage of the SARS-CoV-2 genome from samples containing low template concentrations, and its reliability for high-throughput sequencing. This protocol could be easily applied for the monitoring of other viruses from wastewater, providing cost-effective and population-wide genomic surveillance during disease outbreaks.

## Supporting information

S1 File Step-by-step protocol, also available on protocols.io. (PDF)

S2 File Synthetic SARS-CoV-2 RNA variant mixtures and serial dilutions. (XLSX)

S3 File R scripts used to generate figures. (TXT)

S4 File List of ENA sample accessions and sample IDs associated with each figure. (CSV)
