# Supplementary material for: Optimised protocol for monitoring SARS-CoV-2 in wastewater using reverse complement PCR-based whole-genome sequencing
Source: PLoS One. 2023 Apr 14;18(4):e0284211. doi: 10.1371/journal.pone.0284211 (PMC10104291; doi:10.1371/journal.pone.0284211)
Supplement: S1 File — (PDF) [file pone.0284211.s001.pdf]

protocols.io

Protocol for sequencing SARS-CoV-2 from wastewater

Version 3.0

Authors: Harry T. Child (University of Exeter), Paul A. O'Neill (University of Exeter), Karen Moore (University of Exeter), Hubert Denise (UK Health Security Agency), Matt Loose (University of Nottingham), Steve Paterson (University of Liverpool), Ronny van Aerle (Cefas), Aaron R. Jeffries (University of Exeter)

Centre for Environment Fisheries & Aquaculture Science

University of Exeter

University of Liverpool

University of Nottingham UK | CHINA | MALAYSIA

UK Health Security Agency

Version control history

Version	Author	Date	Comment
1.0	A. Jeffries, S. Paterson, M. Loose & R. Van Aerle	17/09/21	Final V1.0
**2.0**	A. Jeffries, H. T. Child, S. Paterson, M. Loose & R. Van Aerle	07/03/22	Final V2.0
**3.0**	Harry T. Child, Paul A. O'Neill, Karen Moore, Hubert Denise, Matt Loose, Steve Paterson, Ronny van Aerle & Aaron R. Jeffries	09/12/22	Final V3.0

Summary

This SOP describes the procedure for generating cDNA libraries from SARS-CoV-2 viral nucleic acid extracts from wastewater samples using an adaptation of the Nimagen EasySeq™ SARS-CoV-2 Protocol v4.01 (https://www.nimagen.com/gfx/Covid19/protcol_NimaGen_covid_wgs_v401.pdf).

Version 2.0 of this protocol is an update to accommodate the change to using lyophilized indexes in the EasySeq™ RC-PCR SARS CoV-2 (novel coronavirus) Whole Genome Sequencing Kit (NimaGen, SKU: RC-COV096). Updates to the SARS-CoV-2 WGS Panel probe mixes have also been made (currently v4.02) to improve coverage of new dominant variants of concern (VoCs), although these can be used with no change to this protocol. For the latest BED files containing primer details for analysis, check the download section of https://www.nimagen.com/covid19. Version 3.0 includes a minor update to the options for post PCR cleanup, following optimization of the composition of the AmpliClean™ beads provided with the kit by NimaGen. Additionally, advice is provided for sequencing poor quality libraries resulting from a high proportion of SARS-CoV-2 negative samples.

The first step of this protocol is an addition to the original method, which aims to remove any PCR inhibitors from extracted RNA samples and potentially concentrate the RNA further, before carrying out reverse transcription to cDNA.

During the second step, which follows the original EasySeq™ SARS-CoV-2 Protocol v4.01, with some minor modifications, reverse complement PCR is performed using a panel of probes that will generate tiled amplicons covering the whole SARS-CoV-2 genome.

The final step involves an improved library clean up that will remove unused/unincorporated PCR probes, which will be more abundant in samples that contain low viral RNA concentrations such as those from wastewater.

1. Reagents

Note: All reagents used should be molecular biology grade (MBG) and all plasticware should be RNase and DNase free.

- Mag-Bind® TotalPure NGS beads (Omega Bio-Tek, SKU: M1378-01) or AMPure RNA XP beads (Beckman Coulter Agencourt, A63881)
- MBG ethanol
- MBG water
- LunaScript® RT SuperMix Kit (New England Biolabs, E3010L)
- EasySeq™ RC-PCR SARS CoV-2 (novel coronavirus) Whole Genome Sequencing Kit v4.0 (NimaGen, SKU: RC-COV096)
- PhiX Control V3 (Illumina, FC-110-3001)

2. Cleanup of RNA Extracts

Aim: Reduction of any PCR inhibitors and potential concentration of RNA. Starting volume is 20 µl but this can be increased if further concentration is required (although for the latter tests on whether inhibitors are also concentrated have not been undertaken).

Notes: Use either AMPure RNA XP beads (Beckman Coulter Agencourt) or Mag-Bind® TotalPure NGS beads (Omega Bio-Tek), the latter being cheaper to use. Use aliquoted beads rather than directly from stock bottle to minimize risk of stock contamination. Ensure the aliquot is at room temperature and thoroughly mixed (vortexed) before use.

PCR Plates – Any style (skirted, non-skirted, full height, low profile), providing they fit the magnetic rack effectively.

Working environment – Ensure you work in a dust free environment, either a dust free bench or PCR cabinet. If you are in an area that may have a high risk for contamination, wipe down benches with household bleach (1 – 5%) prior to use. Automation is possible, e.g. Opentrons (allowing at least 7 minutes on magnet).

2.1 Pipette 20 µl of RNA extracts into a 96 well PCR plate (plate 1).

2.2 Add 36 µl of Mag-Bind® Total Pure NGS beads (1.8X volume) and carefully pipette at least 6 times to mix.

2.3 Leave to stand at room temperature for 5 minutes.

2.4 If there is liquid on the side of the wells, cover the plate with a PCR adhesive seal and pulse spin down in a plate centrifuge (with swing out rotor).

Note: A plate centrifuge with a swing out rotor should be used to minimize contamination in case adhesive seals are not sealed properly.

2.5 Place on magnetic rack holder for at least 3 minutes to allow the beads to pellet.

Note: the time taken for the pellets may vary between samples.

2.6 Remove the supernatant. This should be clear and without any beads if possible, although trace amounts of beads can be tolerated. If a large number of beads are taken up, replace the liquid back into the well, wait 20 seconds and then try again.

2.7 Add 80 µl of 80% ethanol v/v in MBG water to each well and leave to stand at room temperature for at least 30 seconds.

2.8 Remove the ethanol, being careful to avoid the pellets. Ideally remove a column (8 channel pipette) or row (12 channel pipette) at a time.

2.9 Repeat steps 2.7 to 2.8 once more.

2.10 Using a P10 or P20 multichannel pipette, remove any remaining ethanol from the bottom of each well.

2.11 Remove the plate from the magnetic rack.

2.12 Add 9 µl of MBG water to each pellet and carefully, but rapidly pipette mix/resuspend the pellet. Repeat this for the entire plate, changing tips between wells.

2.13 Allow to stand for 5 minutes at room temperature.

2.14 While standing, add 2 µl of LunaScript® RT SuperMix to each well of a new PCR plate on a chill block or ice (plate 2).

2.15 Optional - if there are air bubbles or residue on the side of the wells of plate 1, cover with a PCR seal and pulse centrifuge the plate.

2.16 Place plate back on the magnetic rack and allow the beads to pellet for at least 3 minutes.

2.17 Recover 8 µl of the supernatant and add directly into 2 µl of LunaScript® RT SuperMix previously put into plate 2 (Step 2.13), mixing gently 5 times with the pipette.

2.18 Cover plate 2 with a PCR adhesive seal, pulse spin down in a centrifuge and place the plate on a thermal cycler with heated lid.

2.19 Thermal cycler conditions: 25°C for 2 minutes, 55°C for 45 minutes, 95°C for 1 minute and hold at 4°C.

3. NimaGen Reverse Complement PCR (RC-PCR)

Use the EasySeq™ RC-PCR SARS CoV-2 (novel coronavirus) Whole Genome Sequencing kit from NimaGen using a modified version of the manufacturer's protocol, as follows.

3.1 Thaw on ice:

- SARS CoV2 WGS Panel A probe mix (Black cap)
- SARS CoV2 WGS Panel B probe mix (Red cap)
- RC-PCR Probe Dilution Buffer Plus (Blue cap)
- PCR 2x Hotstart HiFi Mastermix (White cap)

3.2 Spin down:

- SARS CoV2 WGS Panel A probe mix (Black cap)
- SARS CoV2 WGS Panel B probe mix (Red cap)

3.3 Vortex for 3 seconds and spin down:

- RC-PCR Probe Dilution Buffer Plus (Blue cap)
- PCR 2x Hotstart HiFi Mastermix (White cap)

Note: The 2x mastermix contains isostabilizers and may not freeze completely, even when stored at -15°C to -25°C. The mastermix may contain precipitates when thawed at +2°C to +8°C. Always ensure that the mastermix is fully thawed and thoroughly mixed before use.

3.4 Take the two IDX PCR plates and cut off the number of strips needed from both plates. Mark the plates with "A" and "B".

Note: Register the indexes used (IDX set / and well position for each sample). Download the index details for setting up Illumina® samplesheets at the download section of https://www.nimagen.com/covid19

Note: For each sample, two PCR reactions are needed (pool A and pool B). Always use the same strip and well position for the same sample, in order to have identical indexes for the sample in both pools. Example: For setting up 24 samples, cut off strip 1-3 from both IDX plates and store the remaining of the plates immediately at -20°C. 2.3

3.5 Prepare in a fresh 2 mL Eppendorf tube the Probe-Polymerase premix A, by combining

- 0.2 µL panel A probe mix per reaction (black cap)
- 0.8 µL RC-PCR Probe Dilution Buffer Plus per reaction (blue cap)
- 10 µL RC-PCR 2x mastermix per reaction (white cap)
- 6 µL MBG water

Make up a mastermix for 10% extra (excess is supplied in the tubes provided).

Repeat the above for panel B probe.

3.6 Quick-spin the PCR plates (A and B), and remove seals carefully.

3.7 Add to each tube of plate A: 17 µL of the Probe-Polymerase premix A.

3.8 Add to each tube of plate B: 17 µL of the Probe-Polymerase premix B.

3.9 Add to the same well position of both plate/strips A and B: 3 µL cDNA.

3.10 Close the tube strips carefully with caps (included in the kit) or plate seals and mix by vortexing 3 seconds.

3.11 Spin the plates to make sure all liquid is in the bottom of the wells.

3.12 Start the RC-PCR program in the thermal cycler(s) and place samples in the cycler when the block is between 60°C and 98°C, close the lid.

4. Library Cleanup

Note: During the initial optimisation of the protocol, post-PCR purification by 0.6x clean-up with Mag-Bind® beads was found to be more effective than 0.85x clean-up with the original beads supplied in the NimaGen EasySeq™ kits and detailed in the NimaGen protocol. However, NimaGen have since optimised the composition of the AmpliClean™ beads supplied, and recent comparison of these with Mag-Bind® beads has shown equivalent performance for wastewater SARS-CoV-2 library preparation. As such, two consecutive 0.85x clean-up steps with AmpliClean™ beads can be substituted for the 0.6x Mag-Bind® clean-ups detailed here. If Ct values are known, it is possible to alter the volume of each sample in each pool for a more equal representation. As wastewater is often represented by poor Ct values, equal volume pooling is routinely performed. However, if you have included SARS-CoV-2 positive controls, or anomalous wastewater samples with a Ct value significantly lower than other wastewater samples, dilution of these samples is strongly recommended to prevent excessive sequencing (use $2^{(\text{wastewaterCt-positivecontrolCt})}$ as a calculation for the dilution amount). Also, see the NimaGen handbook for further information should you wish to balance your pools more effectively.

4.1 Transfer 10 µl of each well of sample plate for primer panel A using an 8 channel pipette into strip tubes. You can use the same tips throughout the whole plate. Then transfer all of this to a labelled Eppendorf. Ensure the contents of the Eppendorf are mixed well by numerous inversions/shaking or brief vortexing. Pulse spin down the tube.

4.2 Repeat step 4.1 for primer panel B (and for any other plates you have).

4.3 In separate 1.5 ml tubes, pipette 240 µl of Mag-Bind® Total Pure NGS beads (or equivalent) and add 400 µl of the appropriate pool (or if required to use lower volumes, use 0.6 x of Mag-Bind Total Pure NGS beads). Mix well by numerous inversions. Leave for 5 minutes at room temperature. This should be done for each plate that has been pooled.

4.4 Pulse spin down the contents and place on a magnetic rack to pellet the beads.

4.5 Once pelleted, carefully remove the supernatant from each tube.

4.6 Add 800 µl of ethanol to each tube. Do not mix contents with the pipette, just let the ethanol sit in the tube, immersing the pelleted beads.

4.7 After waiting for 1 minute, remove the ethanol and add a further 800 µl of 80% ethanol to each tube.

4.8 Wait 1 minute. Then remove the ethanol from each tube, capping the tube after removal.

4.9 Pulse spin the tube. Place back on the magnet. After 15 seconds on the magnet, use a P20 pipette to remove any remaining ethanol from the tubes.

4.10 Remove tubes from magnetic rack and add 100 µl of LowTE (supplied in yellow tube with NimaGen kit). Flick mix tube to resuspend pellet. Allow to stand for 5 minutes at room temperature. If some of the beads appear to be stuck on the side of the tube, do another flick mix after 2 minutes.

4.11 Pulse spin down the tube and place on a magnetic rack.

4.12 Wait for at least 2 minutes for the beads to pellet and recover 100 µl of supernatant.

4.13 Add 60 µl of Mag-Bind® Total Pure NGS beads to the 100 µl of collected supernatant to perform another 0.6x bead cleanup. To do this, repeat steps 3 onwards but with adjusted volumes for beads, supernatant, 500 µl of 80% ethanol and resuspension in 25 µl of LowTE (from NimaGen kit).

4.14 Upon collection of the supernatant, measure the concentration using the Qubit HS DNA kit (or equivalent). Use 1 µl to measure the concentration but ensure accurate pipetting is performed on this low volume.

4.15 Enter the values for primer panels A and B into the NimaGen Loading Calculator for molarity calculations to assist in loading.

4.16 Optional: Check on a fragment analyzer. You should have approximately 450 bp band with very little material below this. Higher molecular weight material is often found with wastewater samples.

Note: The expected peak at 450 bp can appear small relative to the background of larger and smaller fragments if a large proportion of wastewater samples in the plate are negative for SARS-CoV-2 (which can be determined by Rt-qPCR on RNA samples). In this case, library quantification by qPCR, using a kit such as NEBNext® Library Quant Kit for Illumina® (NEB), is recommended to ensure the loading concentration is not overly skewed by the presence of DNA fragments without sequencing adapters.

5. Sequencing of the libraries

Recommended loading density for sequencing the generated libraries is 150-180 pM on an Illumina NovaSeq™ 6000 or 0.9 pM on an Illumina NextSeq™, with 3% PhiX Control V3 (Illumina), or 9 pM on an Illumina MiSeq™ v2 with 5% PhiX Control V3 (Illumina). If library quality is poor due to a high proportion of SARS-CoV-2 negative samples, and concentration determined by Qubit is relied upon in the absence of qPCR library quantification, these loading concentrations may need to be increased to achieve optimal loading density.
